# Changes in Six-Month Prevalence of Circulatory System Diseases among People Aged 20 Years and Older between 2013 and 2018 in Hunan, China

**DOI:** 10.3390/ijerph18052599

**Published:** 2021-03-05

**Authors:** Zhenzhen Rao, Junjie Hua, Ruotong Li, Yanhong Fu, Jie Li, Wangxin Xiao, Jieyi He, Guoqing Hu

**Affiliations:** Hunan Provincial Key Laboratory of Clinical Epidemiology, Department of Epidemiology and Health Statistics, Xiangya School of Public Health, Central South University, Changsha 410078, China; zhenzhenrao19@gmail.com (Z.R.); huajunjie@csu.edu.cn (J.H.); liruotong@csu.edu.cn (R.L.); fuyanhong_CSU@outlook.com (Y.F.); li_jie@csu.edu.cn (J.L.); xiaowangxin@csu.edu.cn (W.X.); hejieyi@csu.edu.cn (J.H.)

**Keywords:** prevalence, circulatory system disease, household interview survey, China

## Abstract

Recent changes in population-based prevalence for circulatory system diseases (CSDs) remain unreported either nationally or locally for China. Data were from the two-round health service household interview survey of Hunan Province, China, in 2013 and 2018. A Rao–Scott chi-square test was performed to examine prevalence differences across socio-demographic variables. The overall age-standardized prevalence of CSDs increased substantially between 2013 and 2018 for inhabitants aged 20 years and older (14.25% vs. 21.25%; adjusted odds ratio (OR) = 1.59, 95% CI: 1.24–2.04). Hypertensive disease was the most prevalent type of CSD, accounting for 87.24% and 83.83% of all CSDs in 2013 and in 2018, respectively. After controlling for other socio-demographic factors, the prevalence of CSDs was significantly higher in 2018 (adjusted OR = 1.40), urban residents (adjusted OR = 1.43), females (adjusted OR = 1.12) and older age groups (adjusted OR = 5.36 for 50–59 years, 9.51 for 60–69 years, 15.19 for 70–79 years, and 12.90 for 80 years and older) than in 2013, rural residents, males and the youngest age group (20–49 years). The recent increase in the overall age-standardized CSD prevalence and the large prevalence disparities across urban/rural residents, sex and age groups merit the attention of policymakers and researchers. Further prevention efforts are needed to curb the increasing tendency and to reduce the prevalence of disparities across socio-demographic groups.

## 1. Introduction

Circulatory system diseases (CSDs) are the leading cause of death and disability-adjusted life years (DALYs) in China [1]. Of all CSDs, cardiovascular diseases (CVDs) are the most common and, thus, have been most frequently studied in recent decades. The CVD-induced death rate was 309.33 per 100,000 in rural areas and 265.11 per 100,000 in urban areas for China in 2018 [2]. By 2030, the number of annual CVD events are forecasted to increase by over half due to the aging population and population growth [3]. Therefore, it is valuable to regularly assess CSD prevalence in this country, especially for CVD prevalence.

One study reported the national prevalence of CVDs over ten years ago (1.44%) using data of the 2007–2008 China National Diabetes and Metabolic Disorders Study [4]. Many other studies reported the prevalence of specific subtypes of CVDs and relevant risk factors related to China, including stroke, dyslipidemia, hypertension, smoking and body mass index (BMI) [5,6,7,8,9], but none reported the overall prevalence of CVDs. Since 2017, the Global Burden of Disease (GBD) study group has annually updated and published the estimates of incidence, prevalence, mortality and other health measures of over 300 diseases and injuries in more than 195 countries and territories [10]. The GBD study group uses hospital-based surveillance data and hospital discharge records to estimate Chinese CVD prevalence [1]. However, hospital-based surveillance data exclude CVD patients who are not admitted to hospitals and, thus, probably underestimate the prevalence rate substantially. In addition, compared to population-based survey data, Chinese hospitalization data were reported to be seriously influenced by the change of social basic medical insurance scheme [11]. Using data of health service household interview surveys conducted in 2013 and 2018, we examined recent changes in overall and subgroup CSD prevalence in Hunan Province, China (Note: because the survey did not specify the category of CVDs, we report the prevalence of CSDs).

## 2. Materials and Methods

### 2.1. Data Source

**Figure 1:**
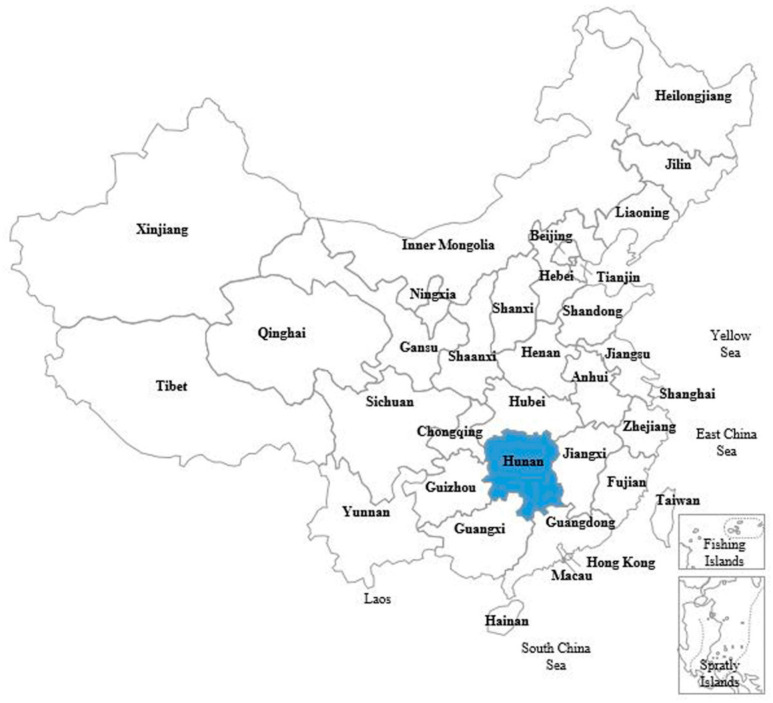
Geographical position of Hunan Province, China.

Data were obtained from the health service household interview survey of Hunan Province, China, administered in 2013 and 2018. The survey was organized by the Health Commission of Hunan Province. Hunan Province is located on the south bank of the Yangtze River and is part of the Central South China region (Figure 1). In 2018, there was a population of approximately 68.99 million residents in Hunan Province [12]. A multi-stage stratified random cluster sampling was adopted to select participants and data were collected through face-to-face household interviews by trained personnel. Details of the survey have been previously published [13,14].

### 2.2. Operational Definition of CSD Event

A chronic disease in this survey was defined if it met any of the following criteria: (a) having a chronic disease that had been clearly diagnosed by doctors within the 6 months prior to when the inhabitants were interviewed; (b) having chronic diseases that had been diagnosed by doctors 6 months ago when the inhabitants were interviewed. Additionally, inhabitants were asked whether they had taken any medication, received physical therapy or received treatment within the 6 months prior to when the inhabitants were interviewed [15].

A CSD event was defined if the respondents reported any of the following 11 kinds of diseases diagnosed by physicians: (1) acute rheumatic fever (I00–I02); (2) chronic rheumatic heart diseases (I05–I09); (3) angina pectoris (I20); (4) acute myocardial infarction (I21); (5) other types of ischemic heart diseases (I22–I25); (6) pulmonary heart disease (I26–I28); (7) other forms of heart disease (I30–I52); (8) hypertensive diseases (I10–I15); (9) cerebrovascular diseases (I60–I69); (10) varicose veins of lower extremities (I83); (11) other and unspecified disorders of the circulatory system (I95–I99) [15,16].

### 2.3. Socio-Demographic Variables

Based on data availability and the relevant literature [2,9,17,18], we included the following variables in our analysis: residence (urban/rural), sex, age group and household income per capita. Age was grouped into seven groups as follows: 20–29, 30–39, 40–49, 50–59, 60–69, 70–79 and ≥80 years.

According to the related previously published studies [19,20], households were equally divided into five categories based on the quintiles of household income per capita in the last 12 months for urban areas and rural areas separately in 2013 and in 2018. The classification criteria for the 2013 survey were as follows: lowest (urban, CNY < 6667; rural, CNY < 3334); low (urban, CNY 6667–9999; rural, CNY 3334–4999); average (urban, CNY 10,000–14,999; rural, CNY 5000–7499); high (urban, CNY 15,000–23,999; rural, CNY 7500–9999); and highest (urban, CNY ≥ 24,000; rural, CNY ≥ 10,000). The classification criteria for the 2018 survey were as follows: lowest (urban, CNY < 10,000; rural, CNY <4500); low (urban, CNY 10,000–15,000; rural, CNY 4500–8333.3); average (urban, CNY 10,000–15,000; rural, CNY 8333.3–13,333.3); high (urban, CNY 22,500–32,500; rural, CNY 13,333.3–20,000); and highest (urban, CNY ≥ 32,500; rural, CNY ≥ 20,000).

### 2.4. Statistical Analysis

A Rao–Scott chi-square test was used to examine the statistical significance of CSD prevalence changes between 2013 and 2018. Prevalence rates were age-standardized using the 2010 China population census. Overall and subgroup analyses were based on age-standardized prevalence rates. Multivariable logistic regression was employed to examine the associations between prevalence rates of CSD and socio-demographic variables. Crude and adjusted odds ratios (ORs) were calculated to quantify the associations. Sampling weights were considered in all statistical analyses. All analyses were conducted with SAS Version 9.4 software (SAS Institute, Cary, NC, USA). Two-tailed *p* < 0.05 was considered statistically significant. 

### 2.5. Ethical Approval

The protocols of the two-round health service household interview surveys were approved by the Hunan Health and Family Planning Commission of Hunan Province, China. Oral consent was obtained for all participants for inclusion before data collection. The protocol of second-hand data analysis for this study was reviewed and approved by the medical ethics committee of Central South University on 24 February 2020 (No. XYGW-2020-46).

## 3. Results

### 3.1. Characteristics of Survey Participants

**Figure 2:**
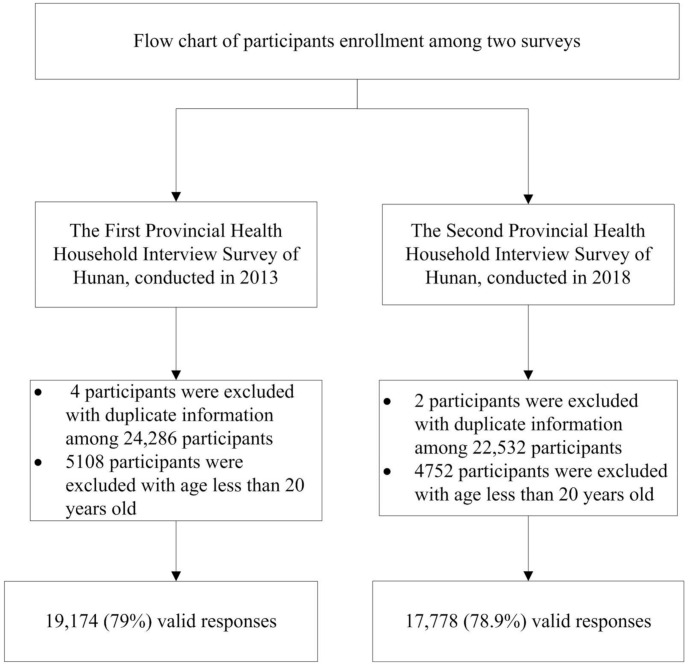
Sample selection procedure of health service household interview surveys of Hunan province, China, in 2013 and 2018.

There were 24,282 and 22,530 residents who completed face-to face interviews in 2013 and 2018, respectively. Among these participants, 79.0% and 78.9% were 20 years and older in 2013 and 2018, respectively (Figure 2). Table 1 shows the characteristics of the survey samples in 2013 and 2018. The composition of the sample did not change significantly between 2013 and 2018 for residence (urban/rural), sex and education but changed significantly for marital status, age group and household income.

### 3.2. Six-Month CSD Prevalence Rate between 2013 and 2018

**Figure 3:**
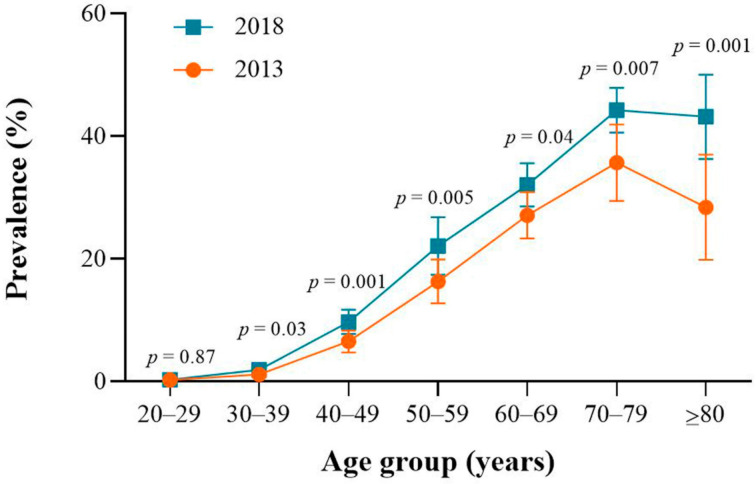
Age-specific 6-month prevalence rates of circulatory system diseases (CSDs) between 2013 and 2018 in Hunan Province, China.

The overall age-standardized CSD prevalence increased substantially between 2013 and 2018 (14.25% vs. 21.25%; adjusted OR = 1.59, 95% CI: 1.24–2.04). Subgroup analysis by residence and sex showed significant increases in rural area (adjusted OR = 1.71, 95% CI: 1.21–2.41), males (adjusted OR = 1.58, 95% CI: 1.13–2.20) and females (adjusted OR = 1.61, 95% CI: 1.29–2.00) (Table 2). The age-specific prevalence rates of CSD increased significantly in five older age groups between 2013 and 2018 (40–49, 50–59, 60–69, 70–79 and ≥80 years; *p* < 0.05) (Figure 3).

### 3.3. Six-Month CSD Prevalence Rate between 2013 and 2018

Table 3 shows that hypertensive disease was the most prevalent CSD subtype. The prevalence of hypertensive disease rose substantially from 12.43% to 17.81% between 2013 and 2018. In addition, hypertensive disease constituted approximately 87% and 84% of all CSDs in 2013 and in 2018, respectively.

### 3.4. Associations between the 6-Month CSD Prevalence and Socio-Demographic Variables

**Figure 4:**
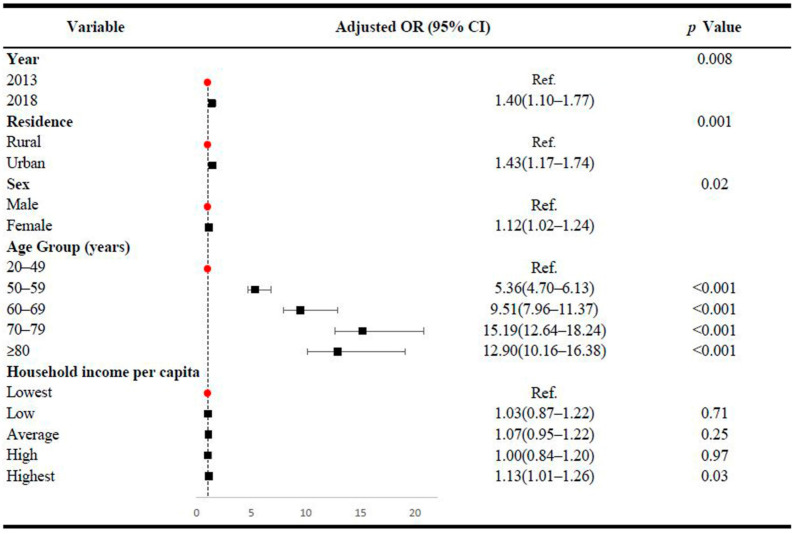
Associations between 6-month prevalence of circulatory system diseases and demographic variables in Hunan Province, China. OR: odds ratio; 95% CI: 95% confidence interval. Adjusted odds ratios were estimated after adjusting for year, residence, sex, age group and household income per capita. Due to small numerators, we combined age groups of 20–29 and 30–39 into a single group, 20–39, to obtain a stable rate. For the 2013 survey, households were divided into five categories: lowest (urban, CNY < 6667; rural, CNY < 3334); low (urban, CNY 6667–9999; rural, CNY 3334–4999); average (urban, CNY 10,000–14,999; rural, CNY 5000–7499); high (urban, CNY 15,000–23,999; rural, CNY 7500–9999); and highest (urban, CNY ≥ 24,000; rural, CNY ≥ 10,000). For the 2018 survey, households were divided into five categories: lowest (urban, CNY < 10,000; rural, CNY < 4500); low (urban, CNY 10,000–15,000; rural, CNY 4500–8333.3); average (urban, CNY 10,000–15,000; rural, CNY 8333.3–13,333.3); high (urban, CNY 22,500–32,500; rural, CNY 13,333.3–20,000); and highest (urban, CNY ≥ 32,500; rural, CNY ≥ 20,000).

Figure 4 demonstrates the multivariate analysis results between CSD prevalence and socio-demographic variables. After adjusting for other socio-demographic factors, the prevalence of CSD was significantly associated with year, urban/rural residence, sex and age group. The prevalence rate was higher in 2018 (adjusted OR = 1.40, 95% CI: 1.10–1.77), urban area (adjusted OR = 1.43, 95% CI: 1.17–1.74), females (adjusted OR = 1.12, 95% CI: 1.02–1.24), inhabitants aged 50–59 years (adjusted OR = 5.36, 95% CI: 4.70–6.13), 60–69 years (adjusted OR = 9.51, 95% CI: 7.96–11.37), 70–79 years (adjusted OR = 15.19, 95% CI: 12.64–18.24) and aged 80 and older (adjusted OR = 12.90, 95% CI: 10.16–16.38) compared to in 2013, rural area, males and inhabitants aged 20–49 years (Note: the test of multicollinearity showed that the multivariable model had no significant multicollinearity).

## 4. Discussion

### 4.1. Primary Findings

Using data from two-round health service household interview surveys in the Hunan Province of China, we updated the recent CSD prevalence rates of residents aged 20 years and older and examined changes in overall and subgroup CSD prevalence rates. First, the overall and certain subgroup age-standardized CSD prevalence rates increased substantially between 2013 and 2018. Second, hypertensive diseases were the most prevalent CSD subtype. Third, CSD prevalence was significantly associated with year, urban/rural residence, sex and age group.

### 4.2. Interpretation of the Findings

Compared with previously published research, our study presents up-to-date population-based CSD prevalence data for Chinese inhabitants aged 20 years and older. The increases in overall crude and age-standardized prevalence rates of CSDs were somewhat different from the GBD 2019 estimates for CVDs among Chinese people aged over 20 years in the same time period directionally (crude prevalence: from 9.1% to 10.3%; age-standardized prevalence: from 6.1% to 6.2%) [21]. The inconsistency might be attributed to differences in the kinds of CSDs, regional characteristics of the Hunan Province compared to the whole country and different data sources.

The recent prevalence increase results were primarily due to the combined effects of elevated prevalence of risk factors, increased routine medical examination and improved diagnosis. During recent years, the prevalence of major risk factors of CSDs has risen rapidly in China. According to recent data from the China Health and Nutrition Survey (CHNS), conducted in 2015 and 2019, the prevalence of high blood pressure among Chinese adults was 27.2%, a continuous increase from the 25.2% reported in a previous survey in 2012 [22,23]. The age-standardized prevalence of obesity and overweight in Chinese adults changed from 19.3% in 2013 to 25.6% in 2018 [24]. In 2018, over 50% of males were tobacco users although the prevalence of smoking among adults over 15 years of age declined slightly from 28.1% to 26.6% between 2012 and 2018 [25,26]. The proportion of residents aged 15 years or older undergoing medical examination was reported to increase from 18.3% to 43.3% between 2008 and 2013 [27]. In addition, a recent study reported that the prevalence rate of hypertension was much higher among Chinese residents undergoing medical examinations than among those not undergoing medical examinations [28]. The increasing use of CT angiography in routine medical examinations by Chinese hospitals is another possible factor of recent increases in CVD morbidity [18,29].

Our findings indicated that hypertensive diseases were the most prevalent CSD subtype. Some studies treated hypertension as a major risk factor for CSDs [2,4,30]. The growing prevalence of hypertensive diseases is probably related to the rise in obesity, physical inactivity and changes in diet among Chinese adults. From 2000 to 2014, the prevalence of obesity and overweight increased from 8.6% to 12.9% and from 27.4% to 41.2%, respectively [31]. In contrast, overall physical activity has been declining, which was largely due the reduction in occupational physical activity. From 1991 to 2011, occupational physical activity fell from 382 metabolic equivalent of task (MET) to 264 MET hours per week in men and fell from 420 MET hours per week to 243 MET hours per week in women [32]. Moreover, the diet pattern among Chinese population has changed significantly. The diet consumption has changed from starchy foods to animal-based for the Chinese. From 1961 to 2017, the per capita calorie intake of red meats increased by 16 times [33].

Our results replicated previous reports on associations of CSD prevalence with residence (urban/rural) and age group. Higher CSD prevalence among urban residents was mainly related to high exposures to major risk factors compared to that for rural residents. For example, urban residents generally face higher psychological stress, higher intake of dietary fat, more sedentary time as well as less exercise time than their rural counterparts [34,35,36,37,38]. In addition, people living in urban communities had better access to medical care and health services, which might cause more early detection of CSD cases in urban areas than in rural areas, especially for those with minor symptoms [39,40]. In contrast with previous studies, our results showed a higher CSD prevalence in females. The difference in prevalence gaps between males and females was probably associated with a higher proportion of females undergoing medical examinations than males, indicating that females with minor or mild symptoms of CSDs were more likely to be detected and treated early [28]. It is normal that CSD prevalence increases rapidly as age increases since almost all physical functions begin to weaken for middle-age adults [41].

### 4.3. Policy Implications

Our study has some important implications. First, our study highlights the importance of increasing investments in CSD prevention and care in Hunan Province. Residents in Hunan Province tend to eat spicy and salty foods. It is worthwhile to promote more and stronger intervention measures, including encouraging regular exercise and a healthy diet in Hunan Province. Second, routine medical examination and standardized management of patients should be promoted for early detection and treatment of CSDs. Third, our results imply that additional actions for key populations should be targeted by the government to reduce inequities among different population groups. Last, further research is needed to interpret the observed increase in prevalence and explore new intervention methods to improve CSD prevention and control.

### 4.4. Limitations of This Study

This study was primarily limited by data availability and sample size. For this reason, we did not examine changes in mortality and prevalence of relevant risk factors (e.g., smoking, drinking, physical activity and unhealthy diet). Therefore, we cannot obtain robust confidence intervals of the prevalence rate for specific CSDs. Second, face-to-face interviews may cause recall bias for inhabitants with minor or mild symptoms to some extent. In addition, due to lacking identifiable information to match the participants of two-round surveys, we were unable to identify participants who were sampled twice and therefore cannot perform a longitudinal data analysis.

## 5. Conclusions

CSD prevalence rates increased substantially from 2013 to 2018 for inhabitants aged 20 years and older in Hunan Province, China. Hypertensive diseases were the most prevalent CSDs. The prevalence of CSDs was significantly higher in 2018, urban residents, females and older age groups. Future studies are needed to explore risk factor changes for the prevention of CSD and other non-communicable diseases. Further intervention efforts, including promoting healthy lifestyles and increasing routine medical examination, should be implemented to curb the increasing tendency and to reduce the prevalence disparities across socio-demographic groups.

## Figures and Tables

**Table 1:** Characteristics of participants in health service household interviews of 2013 and 2018 in Hunan Province, China.

Characteristics	2013		2018		*p* Value
	Number	Percentage (95%)	Number	Percentage (95%)	
**All participants**	19,174	100.00	17,778	100.00	
**Residence**					
Urban	9816	23.48 (4.57–42.40)	9103	30.61 (6.27–54.95)	0.70
Rural	9358	76.52 (57.61–95.43)	8675	69.39 (45.05–93.73)	
Sex					
Male	9390	48.94 (48.19–49.69)	8642	48.10 (47.21–49.00)	0.13
Female	9784	51.06 (50.31–51.81)	9136	51.90 (51.00–52.79)	
**Age group (years)**					<0.001
20–29	2235	12.19 (10.14–14.23)	1455	7.42 (6.68–8.16)	
30–39	2516	12.34 (10.88–13.79)	2295	11.48 (9.51–13.44)	
40–49	4387	23.49 (21.54–25.45)	3295	17.95 (17.25–18.65)	
50–59	4153	21.73 (18.78–24.69)	4401	26.01 (24.14–27.88)	
60–69	3571	18.07 (15.96–20.18)	3891	23.41 (21.76–25.06)	
70–79	1745	9.05 (7.16–10.94)	1901	10.04 (8.77–11.31)	
≥80	567	3.13 (2.40–3.86)	540	3.69 (2.48–4.90)	
**Education**					
College or above	1952	11.74 (8.86–14.61)	1509	8.59 (6.25–10.93)	0.60
Technical School	5602	32.01 (26.12–37.90)	5321	30.18 (24.23–36.13)	
Senior High school	6386	33.58 (31.14–36.03)	5923	32.95 (31.54–34.36)	
Junior High School	2475	10.84 (7.86–13.82)	2289	13.47 (10.14–16.80)	
Elementary	911	4.36 (2.82–5.91)	930	5.09 (3.59–6.59)	
Illiteracy	1848	7.47 (3.46–11.48)	1806	9.72 (5.46–13.97)	
**Marital status**					0.001
Single	1376	7.01 (5.81–8.20)	948	5.09 (4.19–5.99)	
Married	16,121	84.20 (82.49–85.90)	15,214	84.71 (82.70–86.72)	
Widowed	1376	7.51 (6.49–8.52)	1291	8.16 (6.74–9.59)	
Divorced	283	1.22 (0.84–1.60)	305	1.84 (1.38–2.31)	
**Average income per capita ^a^**					0.008
Lowest	3756	15.70 (12.08–19.32)	3614	17.26 (15.07–19.45)	
Low	2736	11.91 (10.12–13.70)	3592	17.69 (14.30–21.07)	
Average	4284	22.87 (19.68–26.06)	4029	23.26 (20.51–26.02)	
High	3499	16.86 (13.77–19.95)	3020	16.49 (14.09–18.88)	
Highest	4845	32.67 (26.65–38.68)	3508	25.30 (20.01–30.60)	

95% CI: 95% confidence interval. ^a^ For the 2013 survey, households were divided into five categories: lowest (urban, CNY <6667; rural, CNY <3334); low (urban, CNY 6667–9999; rural, CNY 3334–4999); average (urban, CNY 10,000–14,999; rural, CNY 5000–7499); high (urban, CNY 15,000–23,999; rural, CNY 7500–9999); and highest (urban, CNY ≥24,000; rural, CNY ≥10,000). For the 2018 survey, households were divided into five categories: lowest (urban, CNY <10,000; rural, CNY <4500); low (urban, CNY 10,000–15,000; rural, CNY 4500–8333.3); average (urban, CNY 10,000–15,000; rural, CNY 8333.3–13,333.3); high (urban, CNY 22,500–32,500; rural, CNY 13,333.3–20,000); and highest (urban, CNY ≥32,500; rural, CNY ≥20,000).

**Table 2:** Number of cases and 6-month prevalence rates of circulatory system diseases (CSDs) among inhabitants of Hunan Province, China, in 2013 and 2018.

Variable	2013		2018		Crude OR	Adjusted OR ^a^
	Number of Cases	Prevalence (95% CI)	Number of Cases	Prevalence (95% CI)		
**Overall**	2929	14.25 (11.98–16.51)	3563	21.25 (18.86–23.63)	1.63 (1.28–2.01) **	1.59 (1.24–2.04) **
**Residence**						
Urban	1718	17.23 (12.68–21.77)	1899	22.49 (20.88–24.10)	1.39 (1.00–1.94) *	1.37 (0.99–1.91)
Rural	1211	13.33 (10.73–15.94)	1664	20.70 (17.10–24.30)	1.70 (1.24–2.32) **	1.71 (1.21–2.41) **
**Sex**						
Male	1358	13.87 (10.85–16.90)	1676	20.52 (18.11–22.92)	1.60 (1.19–2.16) **	1.58 (1.13–2.20) **
Female	1571	14.61 (12.84–16.37)	1887	21.92 (19.31–24.54)	1.64 (1.33–2.03) **	1.61 (1.29–2.00) **

95% CI: 95% confidence interval; OR: odds ratio; * *p* < 0.05; ** *p* < 0.01. ^a^ Note: Prevalence rates were age-standardized using the 2010 China population census.

**Table 3:** Top five circulatory system diseases (CSDs) of inhabitants between 2013 and 2018 in Hunan Province, China.

Rank	Type of Disease	2013		2018	
		Prevalence %	Proportion %	Prevalence %	Proportion %
1	Hypertensive diseases	12.43	87.24	17.81	83.83
2	Cerebrovascular diseases	0.61	4.30	1.65	7.76
3	Other forms of heart disease ^a^	0.44	3.06	1.73	3.45
4	Other types of ischemic heart diseases ^b^	0.32	2.25	0.42	1.98
5	Angina pectoris	0.09	0.62	0.08	0.39

^a^ Heart diseases other than acute rheumatic fever, chronic rheumatic heart diseases, angina pectoris, acute myocardial infarction, other types of ischemic heart diseases and pulmonary heart disease [14]. ^b^ Ischemic heart diseases other than angina pectoris and acute myocardial infarction [14].

## Data Availability

Data of this paper can be accessed through a standard application procedure according to local health data-sharing regulation.
